# Genome-wide identification and expression analyses of TCP transcription factor genes in *Gossypium barbadense*

**DOI:** 10.1038/s41598-018-32626-5

**Published:** 2018-09-28

**Authors:** Kai Zheng, Zhiyong Ni, Yanying Qu, Yongsheng Cai, Zhaoen Yang, Guoqing Sun, Quanjia Chen

**Affiliations:** 1 College of Agronomy, Xinjiang Agricultural University, Urumqi, 830052 P. R. China; 2 Biotechnology Research Institute, Chinese Academy of Agricultural Sciences, Beijing, 100081 P. R. China

## Abstract

Sea-island cotton (*Gossypium barbadense*) has drawn great attention in the textile industry for its comprehensive resistance and superior fiber properties. However, the mechanisms involved in fiber growth and development are unclear. As TCP transcription factors play important roles in plant growth and development, this study investigated the TCP family genes in *G*. *barbadense* (GbTCP). We identified 75 GbTCP genes, of which 68 had no introns. Phylogenetic analyses categorized the GbTCP transcription factors into 11 groups. Genomic analyses showed that 66 genes are located on 21 chromosomes. Phylogenetic analyses of *G*. *arboreum*, *G*. *raimondii*, *G*. *hirsutum*, *G*. *barbadense*, *Theobroma cacao*, *Arabidopsis thaliana*, *Oryza sativa*, *Sorghum bicolor*, and *Zea mays*, *Picea abies*, *Sphagnum fallax* and *Physcomitrella patens*, categorized 373 TCP genes into two classes (Classes I and II). By studying the structures of TCP genes in sea-island cotton, we identified genes from the same evolutionary branches that showed similar motif patterns. qRT-PCR results suggested that the GbTCPs had different expression patterns in fibers at various developmental stages of cotton, with several showing specific expression patterns during development. This report helps lay the foundation for future investigations of TCP functions and molecular mechanisms in sea-island cotton fiber development.

## Introduction

TCP proteins are plant-specific transcription factors that play important regulatory roles in plant growth and development in a wide range of plants. TCP is an acronym of the earliest described family members: teosinte branched 1 in *Zea mays*, CYC in *cycloidea*, and proliferating cell factors 1 and 2 (PCF1 and PCF2) in *Oryza sativa*^1^. TCP family members contain a conserved TCP domain consisting of 55–59 amino acids; this region contains a basic secondary helix–loop–helix (bHLH domain) structure, which promotes DNA binding. In addition, TCP family members form homologous or allo-diploid interactions with other proteins through the bHLH domain^1–3^.

TCP transcription factor family members are categorized into two classes (Class I and II) according to their TCP domain. Class I, also known as the TCP-P family, includes PCF1 and PCF2; Class II, also known as the TCP-C family, includes CYC and TB1. The most striking difference between the two subfamilies is that Class I members are missing four amino acids in the basic region, whereas Class II members have a conserved polar-rich amino acid region of about 18 amino acids that forms a hydrophilic alpha-helix R domain^1^. In general, the main function of Class I TCP proteins is to promote cell proliferation in leaves, thereby regulating plant growth and development. Class II proteins play inhibitory roles in cell division in plant development, functioning as negative regulators of leaf growth and positive regulators of aging.

Doebley *et al*.^4^ reported that the maize domestication gene, TB1, inhibits the growth and development of lateral branches, whereas the loss of TB1 gene function causes increased lateral branches in maize. TB1 also contributes to the formation of female inflorescences in maize. The CYC gene is involved in regulating the asymmetry of petals and stamens in snapdragon^5^. PCF1 and PCF2 affect DNA replication and repair as well as chromosomal structure changes by binding to the promoter of the PCNA gene^6^. Many recent studies have investigated Class II TCP proteins. *AtTCP2*, *AtTCP4*, and *AtTCP10* inhibit the development of leaves in *Arabidopsis*. *AtTCP13* inhibits the development of leaves through the regulation of miR319 via the mitogen-activated protein kinase signaling pathway, thereby affecting cell division^6^. TCP members play various regulatory roles in cell elongation and petal symmetry^7,8^, regulation of the circadian clock, embryonic development, and seed germination.

TCP proteins are the only known family of transcription factors in plants, and they play antagonistic and synergistic roles in plant growth and development. As a class of ancient transcription factors, the TCP protein is widely present in multicellular algae and mosses; however, such species have fewer TCP family members^9^. By contrast, gymnosperms and angiosperms have numerous members of the TCP family due to gene duplication during evolution^10–12^. At present, TCPs have been identified in more than 20 species, including *Arabidopsis thaliana*^13^, *O*. *sativa*^14^, *Populus euphratica*^15^, *Lycopersicon esculentum*^16^, *Citrullus lanatus*^17^, *Orchis italica*^18^, *Sorghum bicolor*^19^, *G*. *raimondii*^20^, *G*. *arboreum*^21^, and *G*. *hirsutum*^22^. TCP family members are involved in plant growth and development, seed germination, jasmonic acid synthesis, and the regulation of leaf senescence^23,24^. In addition, they participate in the development of gametes^25,26^ and play important roles in circadian rhythm and defense responses^27^.

Cotton is an economically important crop, and its fibers are a source of natural textile materials. Two types of tetraploid cotton (AADD), *G*. *hirsutum* and *G*. *barbadense*, were formed ~1–2 million years ago. Owing to its slender fiber and high strength, *G*. *barbadense* has become an important raw material for high-grade and special cotton textiles. The textile industry is under intense global economic competitive pressure to produce high-quality cotton fibers and respond to consumer demand for pure cotton textiles. Therefore, improving fiber quality is an urgent task. *G*. *barbadense* fiber is an important resource material for studying cotton quality improvement, resistance inheritance, and heterosis utilization^28^. Genome-wide sequencing of diploid cotton was first completed in *G*. *raimondii*^29^ and *G*. *arboreum*^30^, followed by the recent sequencing of *G*. *barbadense*^31^. These sequences allow the identification of GbTCP genes at the whole-genome level.

TCP genes are bHLH transcription factors that have drawn great attention in recent years. These transcription factors are plant-specific, and they play a major role in the regulation of meristem growth and development. Although TCP transcription factors have been described in *A*. *thaliana*, further characterization is needed. Recent studies have shown that TCP proteins play important roles in the early development of cotton fiber. Overexpression of *GhTCP14* in *A*. *thaliana* changes the level and distribution of auxin, thereby affecting root and epidermal cell initiation and elongation^31^. *GbTCP15* silencing in cotton causes shorter fibers. In addition, this transcription factor plays a role in the regulation of jasmonic acid biosynthesis, reactive oxygen species, calcium channels, and ethylene signaling^32^. However, the molecular mechanisms involved in TCP gene function in sea-island cotton fiber development require further clarification. Moreover, it is urgent to identify the number of TCP genes, their protein structures, and physicochemical properties in sea-island cotton. This study identified the TCP family members in *G*. *barbadense*. In addition, we analyzed the physical and chemical properties and sequence characteristics of TCP proteins. We revealed the expression patterns of TCP genes at various developmental stages in *G*. *barbadense*. The information from this report will be useful for improving cotton fiber quality in the future.

## Results

### Identification of the TCP gene family in *G*. *barbadense*

Members of the TCP transcription family have a conserved domain called the TCP domain^1^. To identify the members of the TCP transcription factor family in *G*. *barbadense*, 115 protein sequences were searched in the database (Supplementary Dataset 6); we excluded proteins without the TCP domain, those containing deletions or non-full-length segments, and those with redundant sequences of the same genes. We identified 75 matched gene sequences that had TCP domains consisting of 54–58 amino acids. Based on the nomenclature of *Arabidopsis* genes, we named the 75 *G*. *barbadense* TCP genes GbTCP1–GbTCP75.

Further analyses of the 75 TCP genes in *G*. *barbadense* revealed that the base length of the coding region of this family ranged between 546 and 1647 bp, with a mean length of 1044 bp. The predicted amino acid length ranged between 181 and 548 amino acids, with a mean length of 347 amino acids. The isoelectric point of the amino acids ranged between 5.8 and 10.28, with a mean value of 8.15. The predicted molecular mass of the proteins ranged between 20 and 58 kDa, with a mean molecular mass of 37.49 kDa (Table 1).

**Table 1:** TCP gene family in *G*. *barbadense*.

GbTCP gene	Gen symbol	Length (aa)	MW (Da)	PI	Nucleotide length (bp)	Chr.Location
GbTCP1	Gbscaffold3209.1.0	398	43667.39	9.12	1197	At_Chr07:3209:117679..119246 (−strand)
GbTCP2	Gbscaffold17189.2.0	410	45042.61	8.76	1233	scaffold 17189:6809..8503 (−strand)
GbTCP3	Gbscaffold13071.3.0	410	44931.16	6.75	1233	scaffold 13071:90196..91428 (+strand)
GbTCP4	Gbscaffold26927.3.0	410	44891.10	6.77	1233	At_Chr05:26927:34102..37231 (−strand)
GbTCP5	Gbscaffold9879.8.0	463	50146.82	7.01	1392	Dt_Chr10:9879:133400..134985 (−strand)
GbTCP6	Gbscaffold10110.20.0	448	48610.09	6.50	1347	Dt_Chr01:10110:264271..267394 (+strand)
GbTCP7	Gbscaffold14699.6.0	446	48341.77	6.52	1341	At_Chr1:14699:173200..176290 (+strand)
GbTCP8	Gbscaffold11947.1.0	401	43857.08	6.23	1206	Dt_Chr05:11947:8102..9639 (+strand)
GbTCP9	Gbscaffold3304.3.0	401	43770.03	6.34	1206	At_Chr4:3304:50754..53349 (−strand)
GbTCP10	Gbscaffold27504.10.0	326	36208.93	5.80	981	Dt_Chr12:27504:134857..137301 (+strand)
GbTCP11	Gbscaffold3032.9.0	325	36033.79	5.80	978	At_Chr12:3032:97502..99904 (−strand)
GbTCP12	Gbscaffold4415.8.0	300	31950.48	7.98	903	Dt_Chr10:4415:151551..153362 (+strand)
GbTCP13	Gbscaffold11062.6.0	257	26578.76	9.51	775	Dt_Chr02:11062:196862..200965 (−strand)
GbTCP14	Gbscaffold9836.1.0	258	26681.92	9.72	777	At_Chr03:9836:7544..9624 (+strand)
GbTCP15	Gbscaffold12374.5.0	255	26400.52	9.66	768	At_Chr13:12375:93696..97512 (+strand)
GbTCP16	Gbscaffold6022.10.0	256	26449.59	9.60	771	scaffold:6022:132362..136420 (+strand)
GbTCP17	Gbscaffold5204.13.0	243	25399.58	9.99	732	Dt_Chr12:5204:275630..276729 (+strand)
GbTCP18	Gbscaffold8038.3.0	243	25288.52	10.07	732	At_Chr12:8038:28929..29660 (+strand)
GbTCP19	Gbscaffold13479.8.0	487	51110.92	7.05	1464	Dt_Chr04:13479:112305..114024 (−strand)
GbTCP20	Gbscaffold13479.9.0	487	51114.90	7.05	1464	Dt_Chr04:13479:115147..116733 (−strand)
GbTCP21	Gbscaffold3670.7.0	487	50995.89	7.38	1464	At_Chr04:3670:218204..220153 (+strand)
GbTCP22	Gbscaffold6358.6.0	338	35465.42	9.08	1017	scaffold:6358:38870..40002 (−strand)
GbTCP23	Gbscaffold6358.4.0	318	33564.41	9.62	957	scaffold:6358:28785..29751 (−strand)
GbTCP24	Gbscaffold6358.5.0	292	30750.21	9.95	879	scaffold:6358:30040..31731 (−strand)
GbTCP25	Gbscaffold3763.19.0	292	30750.21	9.95	879	At_Chr11:3763:159012..159890 (−strand)
GbTCP26	Gbscaffold7158.20.0	345	36301.62	8.32	1038	Dt_Chr08:7158:227528..229399 (−strand)
GbTCP27	Gbscaffold3763.21.0	338	35365.29	9.08	1017	At_Chr11:3763:164134..166435 (−strand)
GbTCP28	Gbscaffold10005.2.0	369	39992.18	7.10	1110	scaffold:10005:27378..29771 (+strand)
GbTCP29	Gbscaffold10005.3.0	369	39933.15	7.10	1110	scaffold:10005:41953..43384 (+strand)
GbTCP30	Gbscaffold5915.15.0	300	31935.46	7.30	903	At_Chr10:5915:172472..175178 (−strand)
GbTCP31	Gbscaffold12010.14.0	501	55920.84	6.67	1506	Dt_Chr12:12010:125213..126718 (−strand)
GbTCP32	Gbscaffold6430.1.0	520	58034.18	6.56	1563	At_Chr12:6430:32168..33784 (+strand)
GbTCP33	Gbscaffold11914.1.0	309	34245.51	8.71	930	At_Chr05:11914:2645..5844 (−strand)
GbTCP34	Gbscaffold3271.1.0	311	34381.65	8.58	936	Dt_Chr04:3271:52752..56167 (+strand)
GbTCP35	Gbscaffold10591.2.0	181	20888.33	6.97	546	At_Chr09:10591:50944..52832 (+strand)
GbTCP36	Gbscaffold6973.22.0	395	42248.17	6.91	1188	At_Chr11:6973:225102..226598 (+strand)
GbTCP37	Gbscaffold8063.4.0	395	42218.14	6.91	1188	Dt_Chr11:8063:48766..50556 (+strand)
GbTCP38	Gbscaffold2445.12.0	409	43120.44	8.48	1230	Dt_Chr07:2445:123806..125786 (+strand)
GbTCP39	Gbscaffold3074.6.0	418	44438.78	8.60	1257	At_Chr07:3075:53199..55192 (+strand)
GbTCP40	Gbscaffold1228.13.0	406	44113.56	6.75	1221	Dt_Chr12:1228:202373..204040 (+strand)
GbTCP41	Gbscaffold12205.15.0	406	44171.60	6.62	1221	Dt_Chr07:12205:408964..410321 (−strand)
GbTCP42	Gbscaffold255.7.0	406	44071.52	6.75	1221	At_Chr12:255:115731..117563 (−strand)
GbTCP43	Gbscaffold1098.5.0	344	37567.58	8.85	1035	Dt_Chr12:1098:91344..92648 (−strand)
GbTCP44	Gbscaffold1098.6.0	344	37607.69	8.85	1035	Dt_Chr12:1098:105220..106451 (−strand)
GbTCP45	Gbscaffold11587.4.0	365	39732.14	9.26	1098	Dt_Chr13:11587:92002..93910 (−strand)
GbTCP46	Gbscaffold11587.3.0	351	38091.43	9.01	1056	Dt_Chr13:11587:84998..86280 (−strand)
GbTCP47	Gbscaffold2634.14.0	351	38091.43	9.01	1056	At_ Chr13:2634:548185..549445 (+strand)
GbTCP48	Gbscaffold2634.13.0	350	37945.38	9.43	1053	At_ Chr13:2634:545016..546265 (+strand)
GbTCP49	Gbscaffold2634.11.0	365	39572.10	9.14	1098	At_ Chr13:2634:524590..526305 (+strand)
GbTCP50	Gbscaffold4159.7.0	266	30312.08	7.78	801	At_Chr07:4159:105843..107385 (+strand)
GbTCP51	Gbscaffold24195.4.0	324	37158.25	8.67	975	Dt_Chr11:24195:40530..42344 (+strand)
GbTCP52	Gbscaffold3298.31.0	325	37548.66	9.05	978	At_Chr11:3298:266275..267753 (−strand)
GbTCP53	Gbscaffold10878.77.0	361	40868.45	7.76	1086	Dt_Chr12:10878:675793..677225 (−strand)
GbTCP54	Gbscaffold20071.10.0	354	40070.77	8.62	1065	At_Chr12:20071:84673..85769 (−strand)
GbTCP55	Gbscaffold8597.2.0	337	36405.77	6.34	1014	Dt_Chr09:8597:1518..3614 (−strand)
GbTCP56	Gbscaffold3493.31.0	341	36933.31	6.26	1026	At_Chr09:3493:413695..414872 (−strand)
GbTCP57	Gbscaffold3493.34.0	341	36933.31	6.26	1026	At_Chr09:3493:426673..427826 (−strand)
GbTCP58	Gbscaffold3276.14.0	384	41042.16	8.95	1155	Scaffold:3276:404854..406503 (+strand)
GbTCP59	Gbscaffold7552.1.0	312	33464.86	9.69	939	At_Chr12:7552:5798..7141 (−strand)
GbTCP60	Gbscaffold4907.6.0	300	31763.48	8.67	903	Dt_Chr04:4907:280558..283567 (+strand)
GbTCP61	Gbscaffold4907.5.0	300	31767.46	8.67	903	Dt_Chr04:4907:278542..279485 (+strand)
GbTCP62	Gbscaffold4840.4.0	298	31463.13	9.49	897	At_Chr09:4840:398461..399357 (−strand)
GbTCP63	Gbscaffold4840.3.0	298	31407.02	9.52	897	At_Chr09:4840:394814..398315 (−strand)
GbTCP64	Gbscaffold8136.6.0	304	32525.09	8.65	915	Dt_Chr07:8136:112863..114487 (+strand)
GbTCP65	Gbscaffold20098.3.0	294	31335.97	9.51	885	Dt_Chr12:20098:60296..62034 (+strand)
GbTCP66	Gbscaffold15344.21.0	211	22717.48	9.05	636	At_Chr13:15344:175451..176086 (+strand)
GbTCP67	Gbscaffold15344.20.0	196	21105.75	8.91	591	At_Chr13:15344:173880..175470 (+strand)
GbTCP68	Gbscaffold12564.5.0	196	21187.85	8.93	591	Dt_Chr13:12564:37197..37911 (+strand)
GbTCP69	Gbscaffold12564.6.0	196	21153.83	8.93	591	Dt_Chr13:12564:41196..41900 (+strand)
GbTCP70	Gbscaffold15617.7.0	548	57784.21	6.72	1647	Dt_Chr01:15617:89104..91488 (+strand)
GbTCP71	Gbscaffold7853.2.0	200	20978.22	10.28	603	At_Chr05:7853:15611..16213 (−strand)
GbTCP72	Gbscaffold34286.3.0	463	50230.89	7.01	1392	At_Chr10:34286:47234..49758 (+strand)
GbTCP73	Gbscaffold12073.3.0	435	48293.95	6.07	1308	Dt_Chr04:12073:264571..267141 (+strand)
GbTCP74	Gbscaffold7711.1.0	344	37621.73	8.53	1035	At_Chr12:scaffold7711:2991..4722 (−strand)
GbTCP75	Gbscaffold354.4.0	302	32016.81	9.81	909	At_Chr12:scaffold354:306918..308284 (−strand)

**Figure 1:**
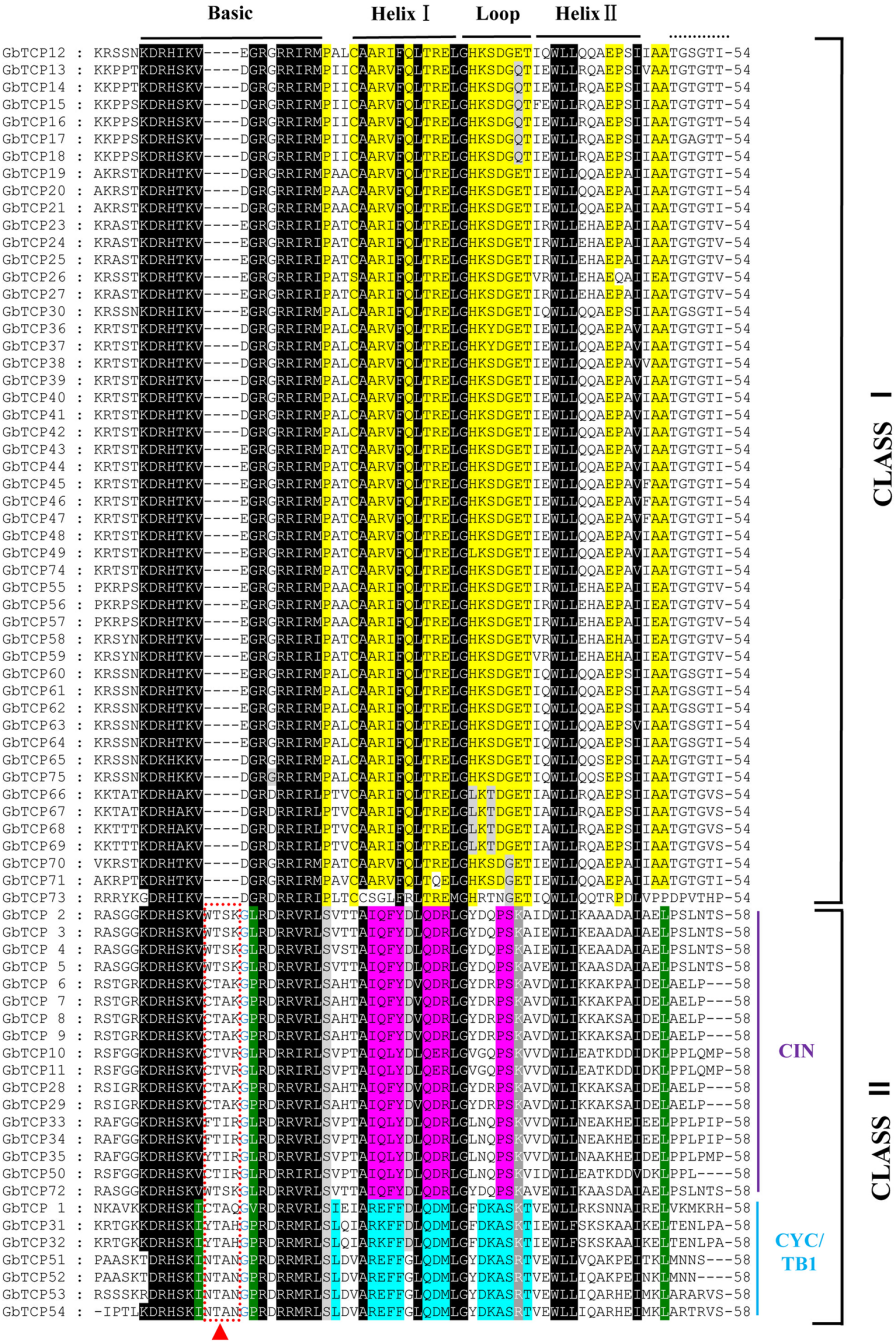
Multiplex sequence alignment of 75 proteins in the GbTCP family. Each letter represents one amino acid, and the left column corresponds to the name of the gene. The black region indicates the highly conserved residues of the GbTCP family members, and the yellow region indicates residues conserved only in the class I subfamily. The purple region indicates residues conserved in the Class II subfamily of CIN-like proteins. Blue indicates the residues conserved in the CYC TB1 class of proteins. The top black bar represents the conserved domain in TCP proteins.

**Figure 2:**
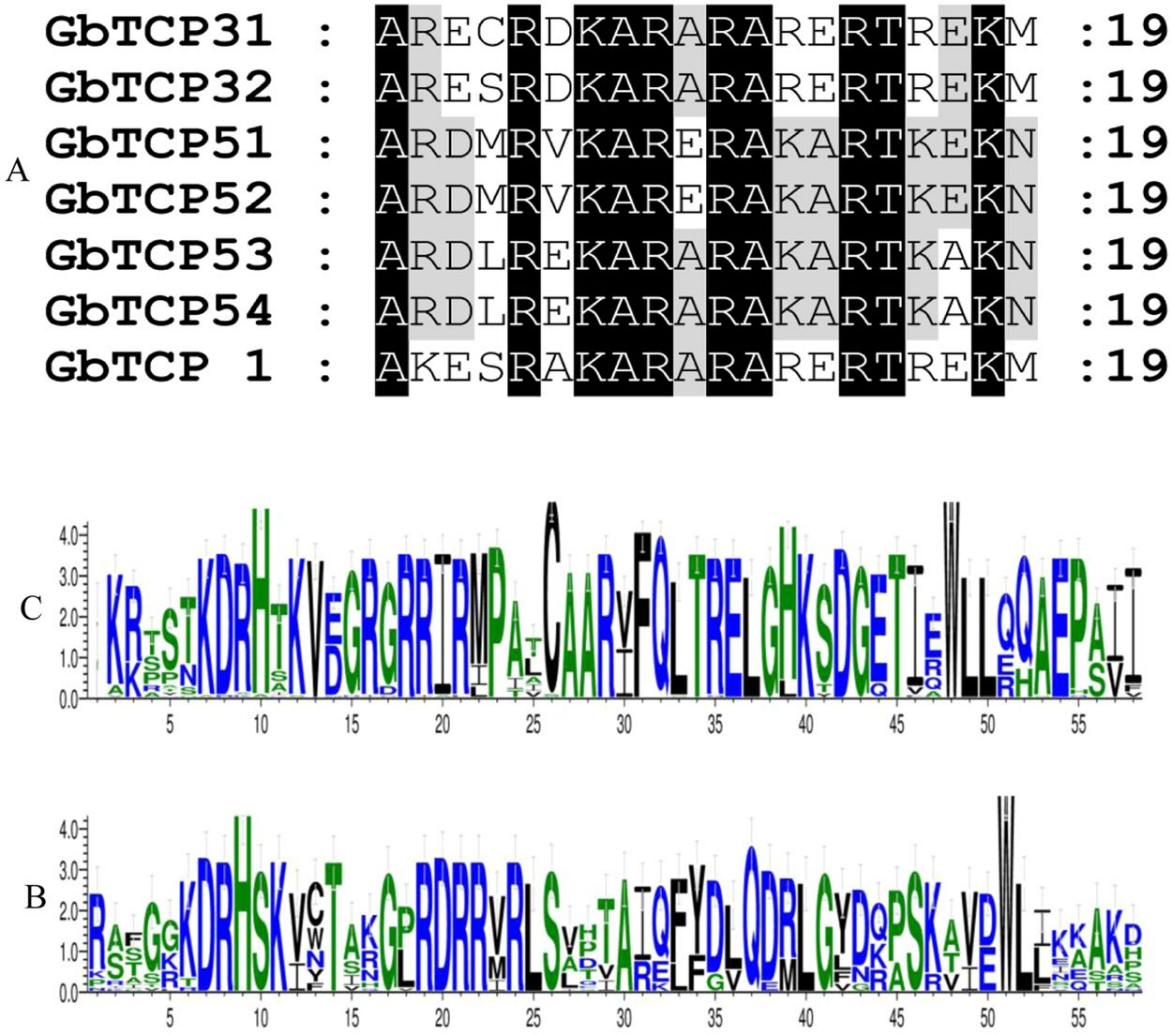
(**A**) The R domain in CYC/TB1 class members of the GbTCP gene family; (**B**) a conserved motif in the Class I subfamily of the GbTCP gene family; (**C**) a conserved motif in the Class II subfamily of the GbTCP gene family.

Analyses of the 75 TCP protein domains of sea-island cotton revealed a typical bHLH domains composed of 54 or 58 amino acids. Figure 1 depicts the entire TCP domain, showing that the conserved rate of the alkaline region was 93.3%, whereas the conserved rate of the ring region was only 62.2%. Based on the TCP domain classification by Cubas *et al*.^1^, the TCP family of sea-island cotton can be divided into Class I and II subfamilies. The most obvious difference between these classes is that Class I members are missing four amino acids in the alkaline region. In *G*. *barbadense*, 50 GbTCP members belong to the Class I subfamily, and 24 GbTCP members belong to the Class II subfamily. In addition to the bHLH structure, the Class II subfamily is subdivided into two branches: CYC/TB1 and CIN. The CYC/TB1 gene has a conserved R domain, which is rich in polar amino acids and forms a hydrophilic α-spiral^1^. Of the TCP genes in *G*. *barbadense*, seven are structurally classified as CYC/TB1 and their R domains consist of 19 amino acids (Fig. 2).

### Evolutionary analyses of the TCP transcription factor family

**Figure 3:**
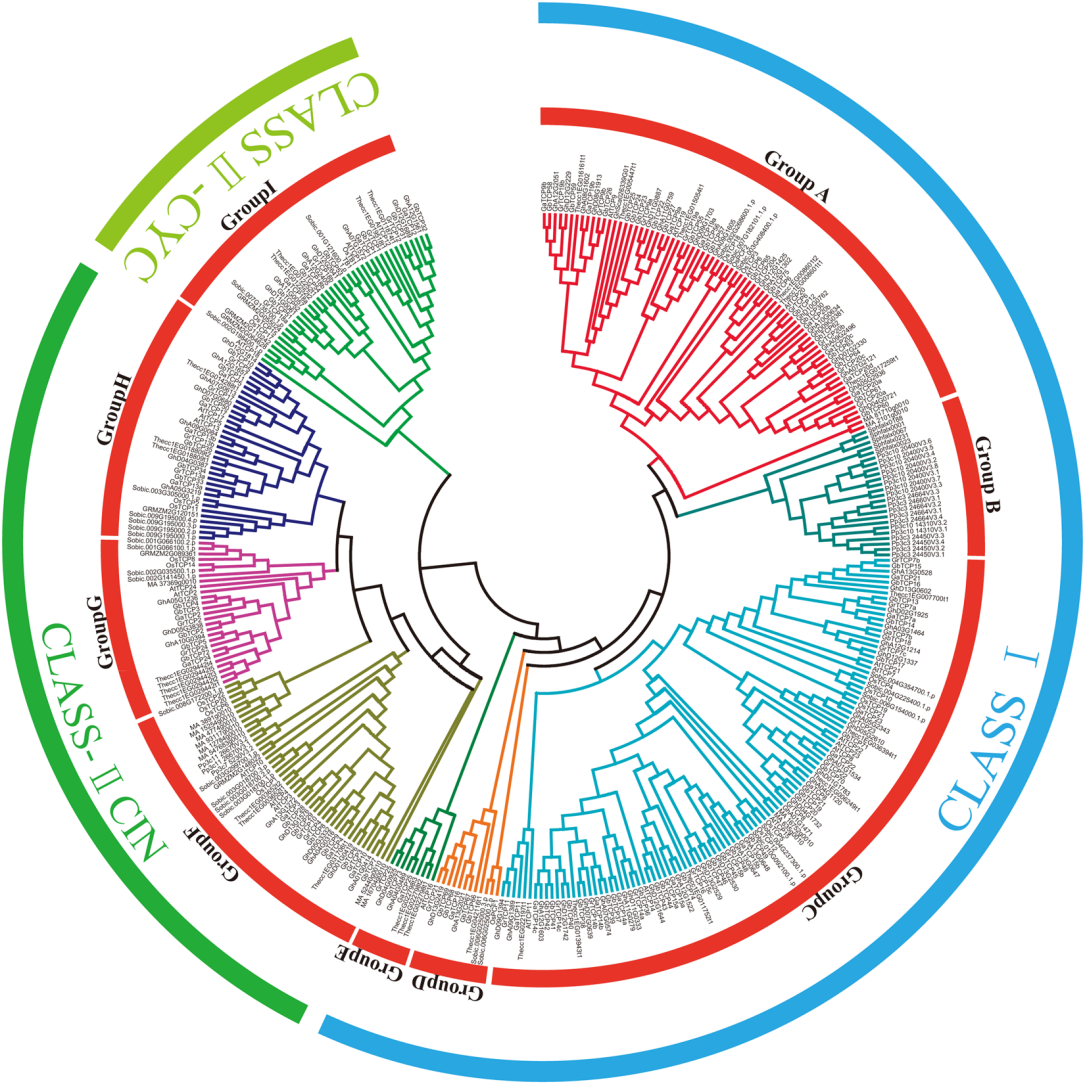
Phylogenetic tree of TCP genes indicating that TCP genes can be clustered into nine groups. Phylogenetic tree of TCP proteins from *Gossypium arboreum*, *G*. *raimondii*, *G*. *barbadense*, *Theobroma cacao*, *G*. *hirsutum*, *Arabidopsis thaliana*, *Oryza sativa*, *Sorghum bicolor*, and *Zea mays*, *Picea abies*, *Sphagnum fallax* and *Physcomitrella patens* using the MEGA6.0 software neighbor-joining method, the JTT model, and a BootStrap set of 1000 repeats for building a rootless tree. The outer circle is marked in blue, green and dark green, which represent the Classes I, Classes I -CIN, Classes II -CYC.

To further investigate the evolutionary relationship of the GbTCP transcription factor family in plants, we investigated the GbTCP sequences in *G*. *arboreum*, *G*. *raimondii*, *G*. *barbadense*, *G*. *hirsutum*, *Theobroma cacao*, *A*. *thaliana*, *O*. *sativa*, *S*. *bicolor*, *and Zea mays*, *P*. *abies*, *S*. *fallax* and *Physcomitrella patens*. These TCP protein sequences were used to construct a tree without roots. Neighbor-joining phylogenetic tree (Fig. 3) analyses divided the TCP transcription factor family into nine different subfamilies, denoted by the letters A–I. The TCP members in *G*. *barbadense* were heterogeneously distributed among nine subfamilies, and the C subgroup was the largest of all subfamilies, with 106 members and 26 GbTCP family members. The smallest branch is that the E subgroup has 9 members, and there is one member of the GbTCP family.

We further classified the TCP proteins in the above plants according to the TCP domain classification methods described by Martíntrillo *et al*.^3^. We categorized 226 members in the A, B, C, D, and E subfamilies that belong to Class I TCP in structure, accounting for 60.5% of the total; 39 members of the I subfamily were structurally categorized into Class II-CYC. A total of 108 TCP members in the F, G, and H subgroups belong to the Class II-CIN category. Further analyses revealed that 51 GbTCP members belong to Class I, accounting for 68% of the total GbTCP family members, and 17 members belong to Class II-CIN, accounting for 23% of the total. Only seven GbTCP members were structurally classified as Class II-CYC, accounting for 9% of the total. Interestingly, except for the algae plant, the TCP genes of these plants were distributed in almost every branch.

### Replication of chromosomes and duplication of genes

**Figure 4:**
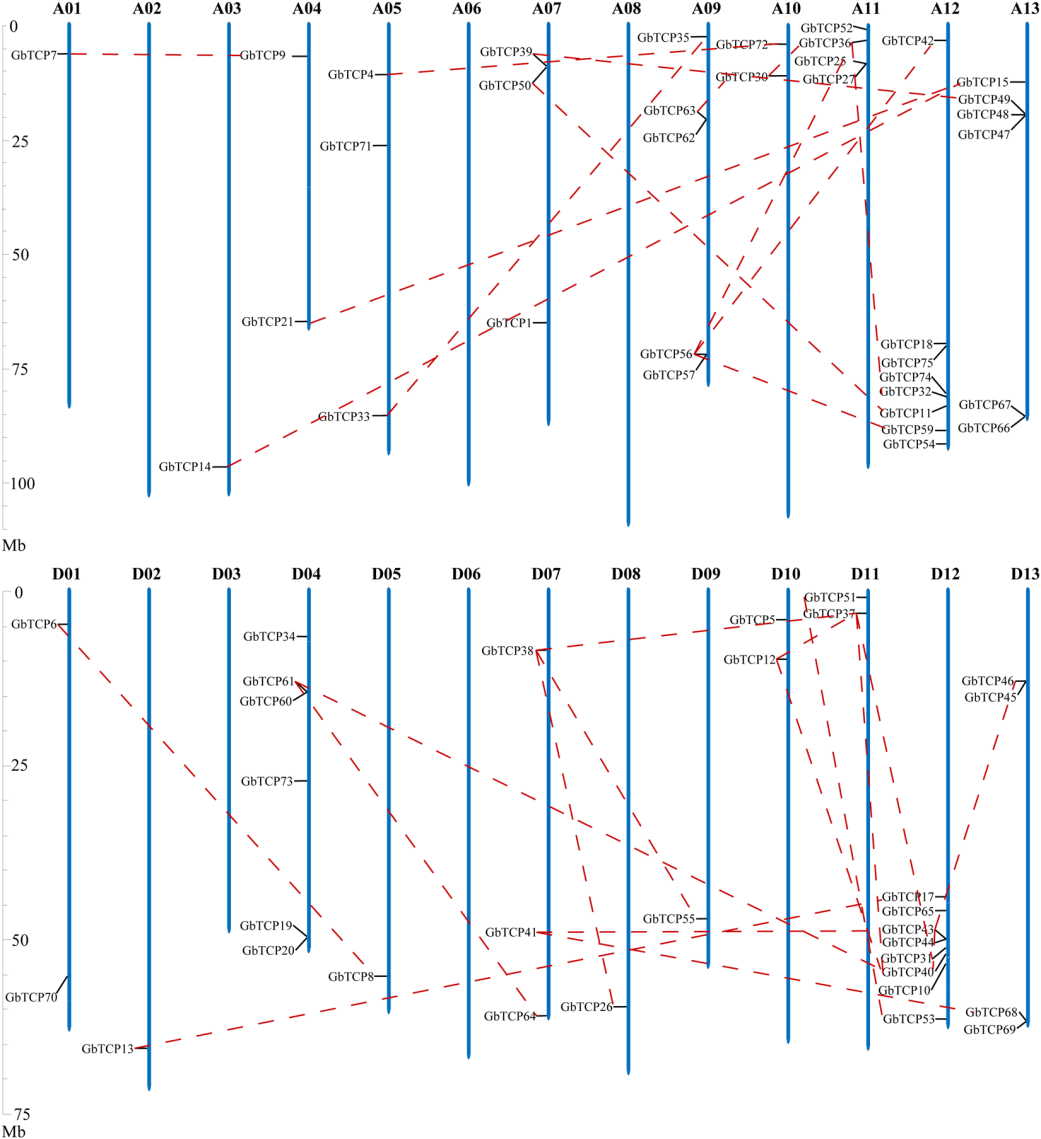
The physical locations of TCP genes on *G*. *barbadense* chromosomes. The red dotted lines link the paralogs TCP genes. The scale is in megabases, Mb.

Analyses of the *G*. *barbadense* TCP gene distribution among the chromosomes showed that 66 GbTCP genes were widely distributed, but non-uniformly, on the *G*. *barbadense* chromosomes; the remaining 9 GbTCPs could not be mapped to any of the chromosomes (Fig. 4). There are no GbTCP genes on chromosomes A02, A06, A08, D03, and D06. The A01, A03, D02, D05, and A03 chromosomes each contain one TCP gene, whereas A04, A10, D01, D10, and D11 chromosomes each contain two GbTCP genes. D13 and A11 each contain four GbTCP genes, whereas chromosomes D04 and A13 each contain six. Most GbTCP genes are concentrated in the A09, A11, A12, A13, D04, D12, and D13 chromosomes. A12 and D12 chromosomes each contain eight GbTCP genes on both ends of the chromosomes.

In the sea-island cotton TCP gene family, seven genes belong to Class II-CYC, and they all have R domains. *GbTCP1* is located on chromosome A7, *GbTCP52* is on chromosome A11, *GbTCP32* and *GbTCP54* are on chromosome A12, *GbTCP51* is on chromosome D11, and *GbTCP31* and *GbTCP53* are located on chromosome D12. These R domain-containing genes show similar distributions on their respective chromosomes, as they are each located near the two ends of the chromosome arms.

Collinearity analyses of gene duplication in the TCP gene family of *G*. *barbadense* were performed as described by Cannon *et al*.^33^. The results revealed that 20 members of the TCP gene family formed 10 tandem replication group pairs, accounting for ~27% of the entire GbTCP family. Two pairs of tandem replication genes were detected on chromosome 9 (*GbTCP62/GbTCP63* and *GbTCP56/GbTCP57*) and chromosome 13 (*GbTCP47/GbTCP48* and GbTCP66/GbTCP67). One tandem replicating genome pair (*GbTCP 25/GbTCP 27*) was located on chromosome 11 of group A. Lastly, there were two tandem replicating genome pairs on chromosome 4 (*GbTCP60/GbTCP61* and *GbTCP1*9*/GbTCP20*) and chromosome 13 (*GbTCP45/GbTCP46* and *GbTCP68/GbTCP69*), and one tandem duplication gene pair (*GbTCP43/GbTCP44*) on chromosome 12 of group D.

**Figure 5:**
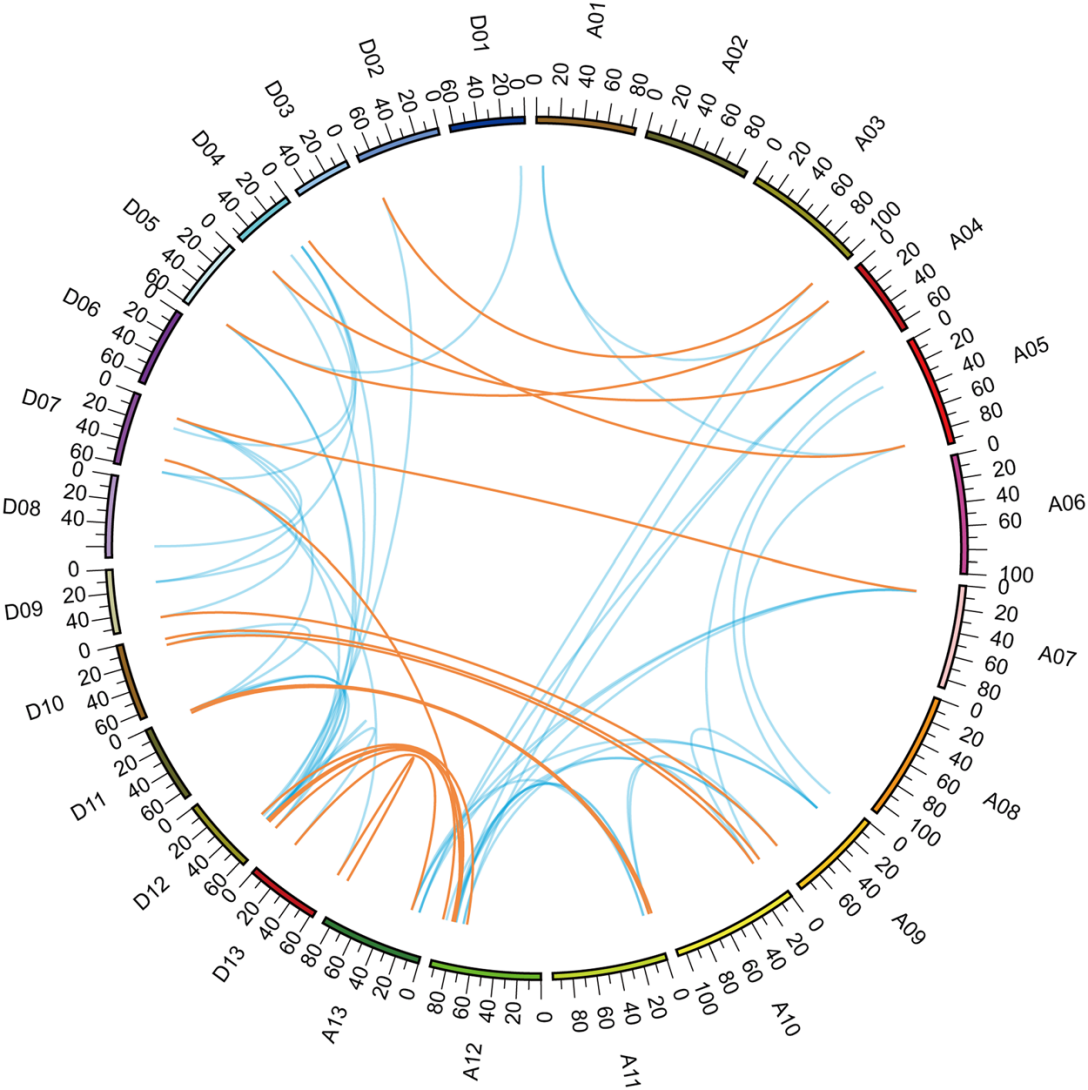
Correspondence between homologous genes in the TCP family of sea-island cotton. The Circos plot shows the relative positional relationship of the TCP genes in sea-island cotton, where each colored band represents a chromosome of *G*. *barbadense*; The ends of the orange lines are oriented toward the orthologous genes from the At and Dt sub-genomes. The ends of the blue lines point toward paralog pairs derived from segmental duplication.

The MCScanX software was used to analyze gene replication and collinearity in the genome segment of the TCP gene family. We identified 42 pairs of TCP genes that have a collinear relationship, some of which are involved in multiple gene duplication events. As shown in Fig. 5, significant collinearity was detected in most TCP members of *G*. *barbadense*. The complex linear relationship indicates that some TCP genes are involved in multiple gene duplication events. This explains why tetraploid cotton has more TCP genes than diploid cotton. In addition, it indicates that TCP gene duplication occurred during the evolution of tetraploid *G*. *barbadense*. We also identified 39 orthologous genes that make up 21 pairs of segment copy pairs (Supplementary Dataset 5), a phenomenon that occurs in orthologous genes. The genes involved are *GbTCP21*/(*GbTCP19* and *GbTCP20*) and *GbTCP67*/(*GbTCP68* and *GbTCP69*). In addition, *GbTCP56*, *GbTCP61*, *GbTCP63*, *GbTCP43*, *GbTCP25*, and *GbTCP19* are involved in both tandem replication and replication of chromosomes. In total, the genes involved in the replication of *G*. *barbadense* include 81.3% of the GbTCP family genes. This indicates that tandem replication and fragment replication play important roles in the expansion of GbTCP family genes in *G*. *barbadense*.

Using the ratio of nonsynonymous substitutions (Ka) to synonymous (Ks) substitutions, we studied the evolutionary selection pressures of TCP genes in *G*. *barbadense*. The results showed that the Ka/Ks ratio of 29 out of 39 paralogs was less than 1 (Supplementary Dataset 4), which indicates that the TCP family of genes in *G*. *barbadense* tend to be purified after chromosomal segment replication.

**Figure 6:**
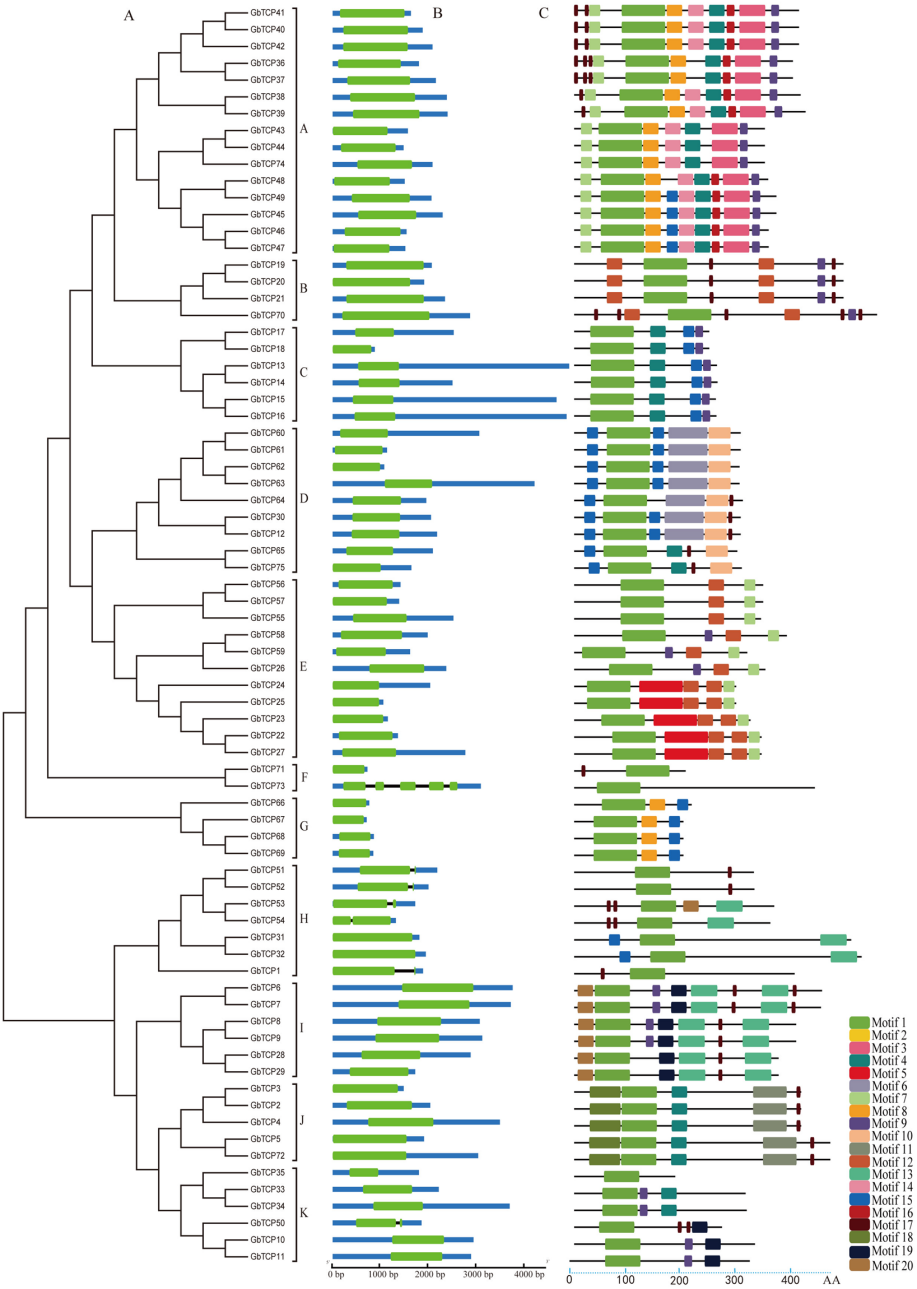
(**A**) Multiple alignment of 75 full-length GbTCP proteins using Clustal 2.0. The phylogenetic tree was constructed using MEGA 6.0 software and linked by the neighbor-joining method with 1000 bootstrap repeats. (**B**) Exon/intron distribution in the GbTCP family genes. Green lines represent exons, and blue lines represent untranslated regions; a scale (bottom) to estimate the size of exons and introns is provided. (**C**) Base sequence diagram of 75 GbTCP family proteins. Each colored box represents a motif of the protein, and the lower scale is used to estimate the size of the protein.

Using the GbTCP family of protein sequences, a neighbor-joining phylogenetic tree was constructed, dividing the GbTCP family member into 11 subfamilies, as shown in Fig. 6A. Next, we used the CDS sequence of the TCP family of *G*. *barbadense* and the gene DNA sequences to further study the intron and exon structures of TCP genes (Fig. 6B). As shown in Fig. 6B, 68 of the 75 GbTCP genes of *G*. *barbadense* had no introns, accounting for 90% of the total. The remaining seven GbTCP genes all contained introns; four in *GbTCP73* and one each in *GbTCP50*, *GbTCP51*, *GbTCP52*, *GbTCP53*, *GbTCP54*, and *GbTCP1*. Compared to several other subfamilies, the two GbTCP genes in subfamily F vary widely in exon length and number of introns. In the GbTCP gene family of *G*. *barbadense*, most of the GbTCP genes in the same subgroup have similar patterns in their exon length and intron number. For example, the GbTCP genes in subfamilies A, B, C, D, E, G, I, and J have no introns; F and K subfamily members have one intron. Analyses of 11 subfamilies of the GbTCP gene family in *G*. *barbadense* revealed that genes generated by repetition have similar gene structure, which suggests that these genes are derived from a common ancestor.

A conserved motif analysis of 75 GbTCP protein sequences in *G*. *barbadense* using the MEME program predicted 20 motifs (Fig. 6C); Subsequently by use the program InterProScan to annotate these motifs. The results show that the only identity that can be identified in the database is the conserved TCP domain (motif1). However, all 75 proteins have a common motif 1. Further analyses within the same subfamily showed that most of the GbTCP members have essentially the same motif composition, but there is a large difference in motif composition among various subfamilies. While the functions of the other 18 sequences are unknown, the conserved motif in the same subfamily of TCP proteins is strikingly similar, which indicates that the structure of GbTCP protein is significantly conserved in a specific subfamily. Interestingly, the subfractions of the F subfamily are spaced far apart, with a low degree of conservation, and a relatively small number of conserved motifs. Compared to other subfamilies, the C and G subfamilies have shorter TCP protein sequences, the motif composition is relatively conservative, and the sequences are significantly reduced. In addition, certain motifs exist only in certain subfamilies. For example, the H subfamily GbTCP genes belong to the TCP–CYC class, but the motif composition in the H subgroup varies greatly from that in other subgroups.

### Analyses of TCP gene expression in *G*. *barbadense* fibers

**Figure 7:**
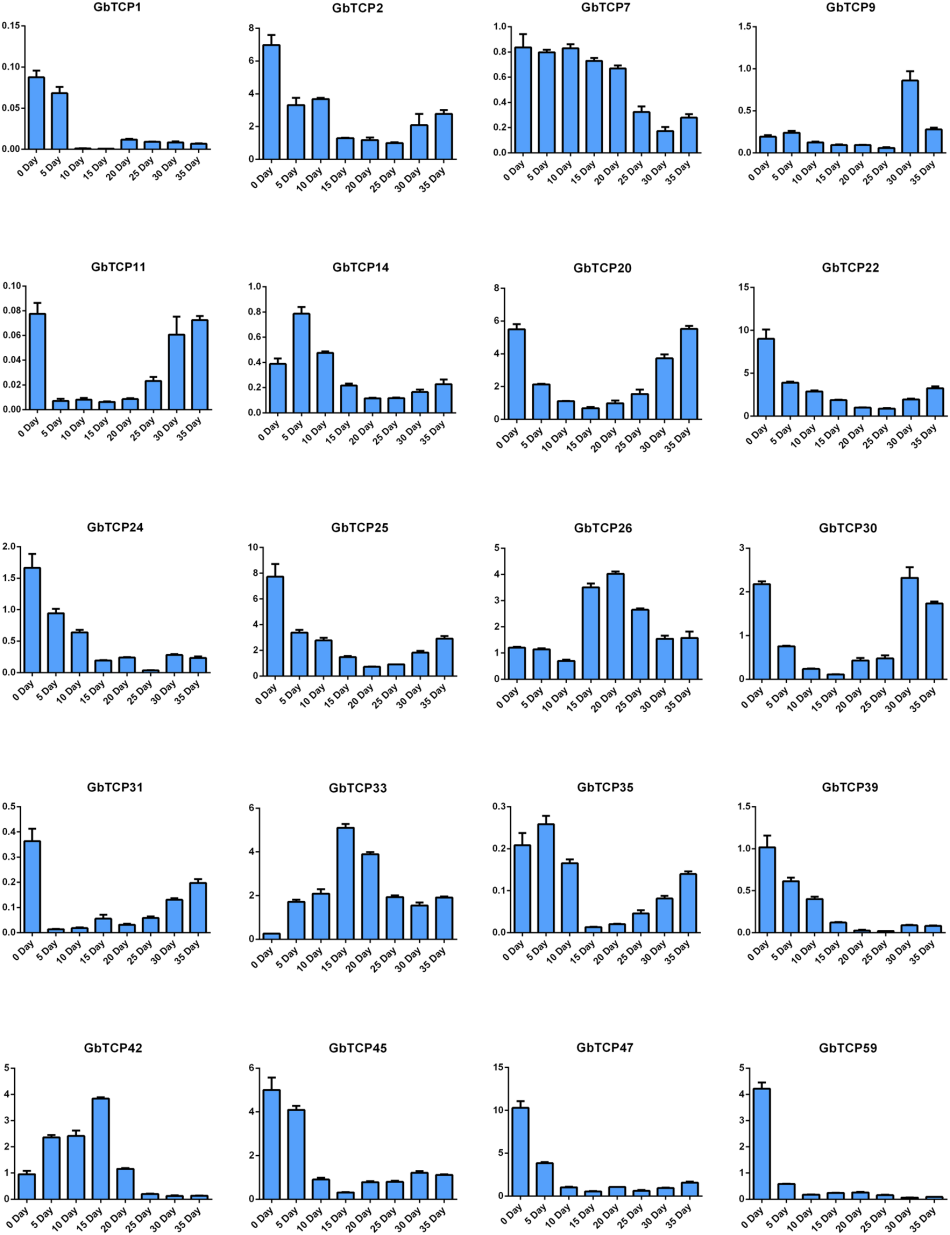
Expression of 20 GbTCP genes in various stages of sea-island cotton fiber development. The X-axis represents fiber samples from different growth periods, and the Y-axis represents the relative expression level of GbTCP gene. Error bars represent the standard deviation of three replicates.

**Figure 8:**
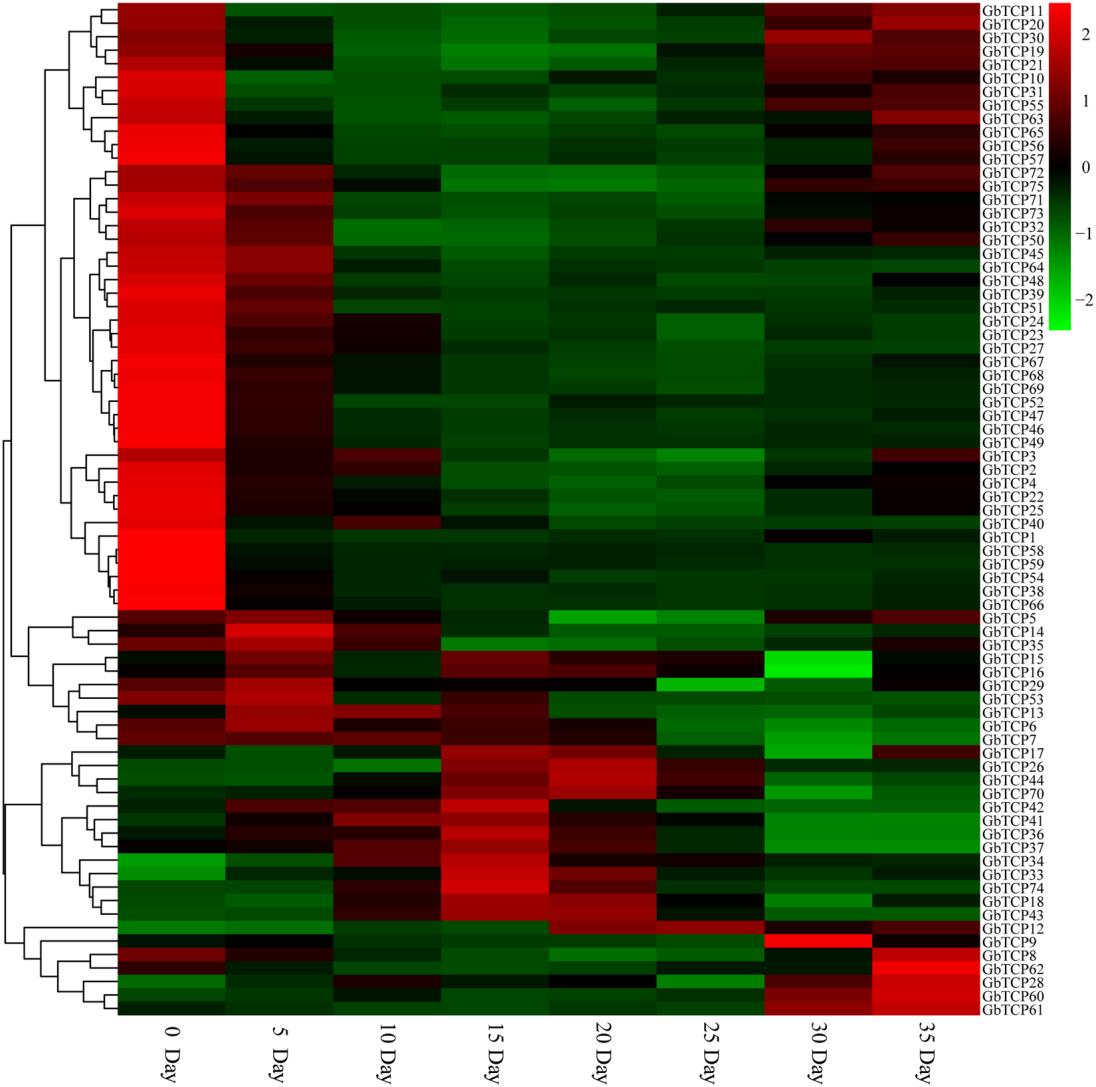
Heat map of the expression patterns of 75 GbTCP genes in various fiber growth stages; expression profile data of GbTCP gene on days 0, 5, 10, 15, 20, 25, 30, and 35 by quantitative real-time (qRT) polymerase chain reaction (PCR). Expression values are log2-transformed. The expression levels are represented by the color bar.

Specific primers were designed to amplify 75 genes in the *G*. *barbadense* TCP family. The expression levels of *G*. *barbadense* (Xinhai 21) TCP genes were analyzed using quantitative real time (qRT)-polymerase chain reaction (PCR) analyses of fiber samples on day 0 (flowering day) and days 5, 10, 15, 20, 25, 30, and 35, with UBQ7 used as the reference gene. As shown in Figs 7 and 8, 48 genes were highly expressed in ovules on day 0 in the initial stage of *G*. *barbadense* fiber development. The *GbTCP17*, *GbTCP26*, *GbTCP44*, *GbTCP70*, *GbTCP42*, *GbTCP41*, *GbTCP36*, *GbTCP37*, *GbTCP34*, *GbTCP33*, *GbTCP74*, *GbTCP18*, and *GbTCP43* genes were highly expressed in fibers on day 15 during the elongation stage of fiber development. *GbTCP12*, *GbTCP26*, *GbTCP44*, and *GbTC*P70 genes were highly expressed in the secondary wall synthesis stage of fiber development. In addition, high expression levels of 16 GbTCP genes were detected at the maturation stage of fiber development. For example, *GbTCP9* was highly expressed in fiber on day 30, and *GbTCP8*, *GbTCP62*, *GbTCP28*, *GbTCP60*, and *GbTCP61* genes were highly expressed in fiber on day 35. No significant differences in expression were detected in *GbTCP1*, *GbTCP2*, *GbTCP3*, *GbTCP31*, or *GbTC39* genes throughout the fiber development period. Furthermore, several *GbTCP* genes showed low expression levels throughout the development period. The varied expression patterns in *GbTCP* genes suggest functional differences in *GbTCP* genes during fiber development.

The gene expression patterns in each branch of the sea-island cotton TCP gene family differ from that in other branches throughout the fiber development period. For example, similar expression patterns were detected in six GbTCP genes (*GbTCP1*, *GbTCP31*, *GbTCP32*, *GbTCP51*, *GbTCP52*, *GbTCP53*, and *GbTCP54*) belonging to the Class II CYC/TBI subfamily. These genes showed high expression only in the ovules on day 0, and low expression during the other periods of fiber development. Analyses of the expression characteristics of 17 genes in the CIN subfamily showed that 12 TCP genes had higher expression in the mature stages of fiber development. High expression of *GbTCP6*, *GbTCP7*, *GbTCP33*, *GbTCP34*, and *GbTCP35* was consistently detected during days 5–20 in cotton fiber development; this period is critical for fibrous cell primary wall elongation. We speculate these five genes may be involved in the fiber cell growth of the primary wall.

We found that Class I TCP gene expression levels differ from Class II gene expression levels in *G*. *barbadense*. *GbTCP* Class I genes have more obvious expression specificity, with expression patterns indicating constitutive expression. For example, *GbTCP17*, *GbTCP26*, *GbTCP36*, *GbTCP37*, *GbTCP41*, *GbTCP42*, *GbTCP44*, and *GbTCP70* all have higher expression in *G*. *barbadense*, whereas *GbTCP40*, *GbTCP58*, and *GbTCP59* genes are expressed only in the ovule. Most of the genes were expressed throughout the entire period, which suggests that some sea-island cotton Class I-TCP genes may be involved in the development of cotton fiber.

### Expression of GbTCPs in Xinhai 25 and Ashmon

**Figure 9:**
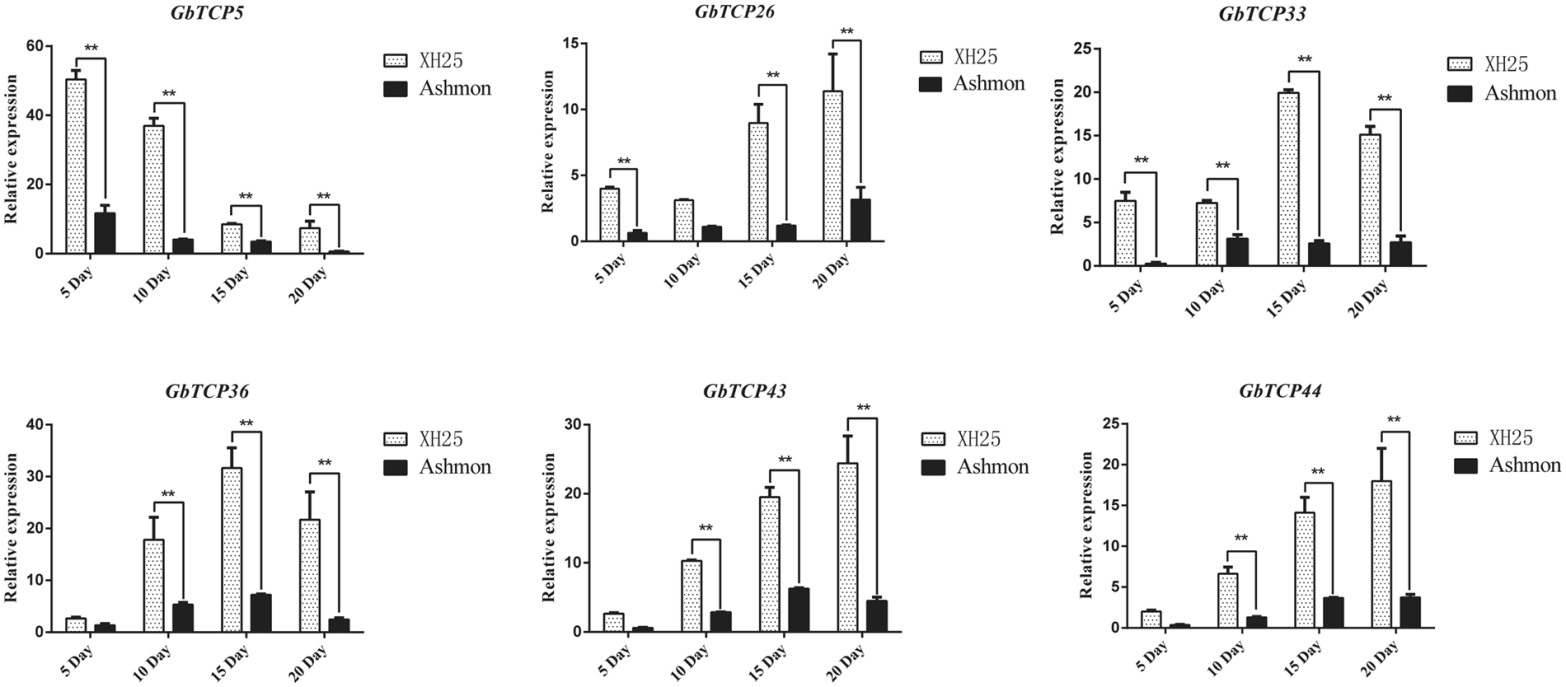
Comparison of the expression of six genes in XH25 and Ashmon. The expression of TCP genes in various fiber developmental stages of Xinhai 25 and Ashmon was analyzed by qRT-PCR; Significant differences between xinhai25 and Ashmon were determined by Student t-test. *Significant differences in Xinhai 25 and Ashmon gene expression levels (P < 0.05); **Greater significant differences in gene expression levels between Xinhai 25 and Ashmon (P < 0.01); Error bars represent SD for three independent experiments. The Y-axis represents the relative expression of genes.

To further study the expression characteristics of GbTCPs in the elongation and synthesis of secondary walls of cotton fiber, we analyzed fiber quality data from the previous 3 years (Supplementary Table 1). Two varieties of cotton with special fiber qualities were selected: Xinhai 25 (longer fibers) and Ashmon (shorter fibers). The fibers from days 10, 15, and 20 were used to study the expression of six GbTCP genes (*GbTCP5*, *GbTCP26*, *GbTCP33*, *GbTCP36*, *GbTCP43*, and *GbTCP44*). As shown in Fig. 9, higher expression levels of six genes were detected in the long fibers of Xinhai 25 than in the short fibers of Ashmon. According to the gene expression trend, made the following classification. It was found that the expression trend of the GbTCP5 gene was different from the other 5 genes. The expression of *GbTCP5* gene was highest in the fiber of 5–10 Day of two cotton varieties, and the expression amount in 5 Day fiber was 6 times as much as that of 15 Day. These findings indicate that *GbTCP5* genes are involved in the initiation stage of cotton fiber development. *GbTCP33* and *GbTCP36* expression increased continuously in fibers from days 5 to 15, peaking on day 15; these findings suggest the involvement of these two genes in the elongation stage of fiber development. The expressions of *GbTCP26*, *GbTCP43*, and *GbTCP44* showed an increasing trend from days 5 to 20, with the highest expression detected on day 20, which suggests the involvement of these three genes in the secondary wall synthesis phase of development.

On this basis, we studied the expression of these six genes in the upland cotton Xinluzhong 36. The results showed that these 6 genes reached their peak values during the 15-day period of fiber development in upland cotton, indicating that these genes play a role in the elongation of the fiber of upland cotton, as shown in the (Fig. 9). In addition, we found that the expression trends of *GhTCP15a-D* (*GbTCP43*) and *GhTCP15a-A* (*GbTCP44*) in 15 days-20 days were different from those in sea-island cotton. The remaining *GhTCP6a-A* (*GbTCP5*), *GhTCP19b-A* (*GbTCP26*), *GhTCP13a-A* (*GbTCP33*) and *GhTCP14a-D* (*GbTCP36*) genes have the same expression pattern in upland cotton and sea-island cotton. At the same time, the expression of these four genes in sea-island cotton was much greater than that of upland cotton. Pearson correlation coefficient was used to detect the correlation between genes in different *Gossypium* species. It was found that these genes were positively correlated in Xinhai 25 and Xinluzhong 36.

## Discussion

The TCP gene family is a class of specific transcription factors that play an important role in plant growth and development by regulating the expression of downstream target genes. However, no systematic studies on the TCP gene family in *G*. *barbadense* have been reported. The TCP gene family in sea-island cotton has many members with complex functions. Moreover, these members can have great genetic differences, displaying various complex, competitive, and interactive relationships. TCP genes in *G*. *barbadense* and *A*. *thaliana* have several differences. First, the number of TCP genes in *G*. *barbadense* is much higher than that in *A*. *thaliana*, which contains 37 more TCP genes than *G*. *raimondii*^20^ and 39 more than *G*. *arboreum*^21^.This finding suggests that the evolution of cotton included diploid to tetraploid gene-gain events. Our results indicate that, compared to the TCP gene family in rice and *Arabidopsis*, the cotton TCP gene family has obvious gene duplication, as evidenced by the great number of genes in the GbTCP family. The phylogenetic tree has numerous and dense branches, but no obvious periphery branches, which indicates that the GbTCP family is fairly conserved. We found that the amino acid lengths in the TCP family members of sea-island cotton differ considerably, which indicates that the origin and evolution of the TCP family of sea-island cottons may be complicated. Furthermore, this variation in length might contribute to the multiple biological functions of TCP genes.

It was found that 7 GbTCP genes in *G*. *barbadense* have a introns, one more than *G*. *raimondii*^20^, three more than *G*. *arboreum*^21^, and three fewer than *G*. hirsutum^22^. This discrepancy may contribute to the more desirable fiber of the sea-island cotton compared to that from other cotton genera. The distribution of introns and exons and the distribution of amino acid motifs revealed that each gene within a subgroup had a similar structure, and that these highly similar sequences are associated with tandem duplication and segmental duplication caused during the evolution of the genome. These phenomena play an important role in the process of genome rearrangement and expansion, as well as in the diversity of gene function and amplification of the gene family^34^.

*G*. *arboreum* is the chromosomal ancestor of the tetraploid sea-island cotton group D, and the *G*. *raimondii* is the ancestor of group A chromosomes. Studying the multiple replication events of the GbTCP gene family will further our understanding of the polyploid formation process in the cotton genome. Large chromosome duplication fragments and tandem duplication of chromosomes play an important role in the amplification of the GbTCP gene family precisely because the GbTCP family of genes tends to be purified after chromosomal segment replication. Therefore, the GbTCP genes are conserved and thus participate in a variety of cotton physiological activities. Structurally, the 10 pairs of tandem duplication genes in sea-island cotton belong to the Class I family. The expression data of different fiber stages revealed these pairs of genes amplified by the same ancestor have the same expression pattern, which indicates that these genes may play similar roles in the evolutionary process.

A previous study reported that a GbTCP transcription factor in *G*. *barbadense* can elongate fibers and root hairs by regulating the metabolism of jasmonic acid and activating downstream genes that control fiber development and root hair elongation^35^. Wang *et al*. found that *GhTCP14* is specifically expressed primarily in the initial stages of fiber development when overexpressed in *A*. *thaliana*. *GhTCP14* overexpression changes the distribution of auxin, and influences the expression levels of auxin-related genes, such as *AUX1*, *PIN2*, and *IAA3*. These findings indicate that *GhTCP14* regulates cotton fiber development by directly regulating auxin^32^.

In this study, the starting point for fiber development occurred on days 0–5, and fiber development extended on days 10–20. In line with those results, *GbTCP36* (GbTCP) and *GbTCP44* (*GhTCP14*) were continuously expressed in the initiation and elongation stages of *G*. *barbadense* fiber development. The expression of *GbTCP36* peaked on day 15 whereas *GbTCP44* peaked on day 20, which indicates that these two genes play an important role in the development of cotton fiber initiation and elongation. *GbTCP5* is involved in the initial stage of fiber development in XH25 and Ashmon. *GbTCP26*, *GbTCP43*, and *GbTCP44* are involved in secondary wall synthesis in fiber development. *GbTCP33* and *GbTCP36* may be involved in fiber elongation and secondary wall synthesis. The expression of structural genes belonging to class A, including *GbTCP37*, *GbTCP41*, *GbTCP42*, *GbTCP43*, and *GbTCP74* (Fig. 6A), show expression characteristics consistent with expression levels reported in *G*. *hirsutum*^22^. However, in the present study, these genes sustained expression in fiber on days 5–20, peaking on day 15. Therefore, *G*. *barbadense* fiber development extends over a long period, which suggests that these genes play an important role in the elongation of *G*. *barbadense* fiber.

TCP genes play an important role as plant-specific transcription factors in plant growth and development. These genes not only regulate cell growth, proliferation, and differentiation they also affect the growth of lateral branches, flowers, and other organs. Studies in *Arabidopsis* have revealed that TCP proteins within the same subfamily share a common motif and have similar functions. Expression of the *AtTCP2*, *AtTCP3*, *AtTCP4*, *AtTCP10*, and *AtTCP24* genes in the CIN subfamily of *A*. *thaliana* is regulated by miR319-mediated post-transcriptional regulation, playing an inhibitory role in cell division during leaf development^36^. The *AtTCP14* and *AtTCP15* genes, which are structural members of the Class I family, are involved in regulating internode development and leaf shape in *A*. *thaliana*, thereby controlling the development of leaves and the proliferation of young internode cells^37^.

The expression characteristics of the GbTCP family in sea-island cotton Xinhai 21 indicated that numerous genes in the GbTCP family are involved in the physiological process of cotton fiber development, and the expression of genes differ in various stages of fiber development. However, genes in the same subfamily showed similar expression trends. For example, GbTCP genes overexpressed in fibers on days 0–5 may be involved in the differentiation and protuberance of fibroblasts. Genes that express significant amounts in fibers on days 10–25 may cooperate with other genes during primary wall elongation and secondary wall thickening. GbTCP genes with high expression on days 30–35 may aid cell dehydration and promote fiber maturation.

This study was the first to identify 75 GbTCP gene family members in *G*. *barbadense* and to investigate their role in cotton fiber development. Our results provide a foundation for future functional studies to determine the molecular mechanisms of TCP genes in the development of cotton fiber.

## Conclusion

We used bioinformatics tools to analyze a genome-wide database to identify 75 TCP genes in *G*. *barbadense*. The GbTCP genes are divided into two subfamilies (Class I and II) according to their structural characteristics; 51 TCP genes belong to Class I, and 24 TCP genes belong to Class II. Chromosomal mapping showed that only 66 of the 75 TCP genes were heterogeneously distributed on 21 chromosomes. Analyses of the collinear relationship among GbTCP genes revealed significant collinear relationships in 81.3% of the TCP genes in *G*. *barbadense*. The complex linear relationship indicated that several TCP genes are involved in multiple gene duplication events. Structural analyses of GbTCP genes in *G*. *barbadense* cotton showed that 68 genes had no introns, and most of the GbTCP genes in the same subgroup had similar patterns of exon length, intron number, and conserved motifs. qRT-PCR analyses of GbTCP gene expression characteristics in the fiber revealed varying expression levels according to the period of fiber development. We identified several genes that are highly expressed in the elongation stage and secondary wall synthesis stage of fiber development, which suggests that GbTCP genes may play an important role in the physiological process of cotton fiber development.

## Materials and Methods

### Materials and growth conditions

*G*. *hirsutum* Xin lu zhong 36, *G*. *barbadense* Xinhai 21, Xinhai 25, and Ashmon were obtained from the Xinjiang Agricultural University, Agronomy Courtyard Key Laboratory of Agricultural Biological Technology. The plants were planted on April 12, 2016, at the Xinjiang Alar City Xinjiang Academy of Agricultural Science Experimental Station. The annual sunshine hours in this location ranged from 2556 to 2991 h, and the average frost-free period was 200 days or more; these conditions were suitable for growth of sea-island cotton. After planting, the crop was maintained under normal field management. Ovules were obtained on the first flowering day, which was marked day 0. Fibers were sampled in duplicate on days 5, 10, 15, 20, 25, 30, and 35. The cotton fibers were placed in liquid nitrogen immediately after sampling for later use.

### Identification of TCP gene family members

The complete genome sequence of sea-island cotton was downloaded from Washington State University in the United States cotton genome database (https://www.cottongen.org/) and the Hua Zhong Agricultural University Center for cotton genetic improvement (http://cotton.cropdb.org/cotton/). Then, we built a local Blast database separately by downloading the TCP hidden Markov model (PF03634) from the Pfam database (http://pfam.xfam.org/) and used it as a BLAST query. The search was performed using HMMER 3.0, and the results obtained were searched using SMART (http://smart.embl-heidelberg.de/) and ExPASy-PROSITE (http://www.expasy.org/) online tools to make structural predictions of the searched proteins. We manually deleted sequences with no TCP domains and non-full-length genes. All remaining TCP amino acid sequences were predicted for molecular weight and isoelectric point using the online ProtParam tool (http://www.expasy.org/protparam/).

### Sequence alignment and phylogenetic analyses

The 24 TCP protein sequences of *A*. *thaliana* were downloaded from the *Arabidopsis* resource database TAIR (http://arabidopsis.org/). The TCP protein sequences of *T*. *cacao*, *O*. *sativa*, and *G*. *hirsutum* were from the plant transcription factor database (http://planttfdb.cbi.pku.edu.cn/). The TCP protein sequences of *G*. *raimondii* and *G*. *arboreum* were from the cotton genome database (https://www.cottongen.org/). We performed multiple sequence alignment of the TCP protein sequences from the above seven plants using ClustalX^38^. We used NJ MEGA 6.0 software^39^ and JTT model for these analyses and the results were a root-free evolutionary tree (execution parameters: BootStrap method 1000; Poisson model: pairwise deletion). To validate the NJ tree, the maximum likelihood (ML) method was also used. The bootstrap method was used with 1,000 replicates.

### Chromosomal location and gene duplication

The chromosomal location information of each TCP gene was downloaded from the sea-island cotton database (http://cotton.cropdb.org/). The 75 genes of the TCP family were located on 26 chromosomes of *G*. *barbadense*, as determined using the MapInspect2.2 software. All the protein sequences of sea-island cotton were included in a local database using Basic Local Alignment Search Tool (BLAST). The entire protein sequences were used as queries to search the above-mentioned database with an e-value of 1e^−5^. The blastp result was analyzed by MCScanX^40^ to produce the collinearity blocks across the whole genome. We used the Circos tool to visualize the chromosomal repeat fragment information and the chromosomal locations of the TCP genes^41^. The replacement rates of synonymous (Ks) and nonsynonymous (Ka) mutations were calculated as described previously^42^. These rates were used to identify DNA polymorphisms using DnaSPV5.0 software^43^, and the Ka/Ks ratio was analyzed to assess the selection pressure of each gene. In general, Ka/Ks > 1 indicates a positive selection effect, Ka/Ks < 1 indicates a purification option, and Ka/Ks = 1 indicates a neutral selection^44^. We use the formula T = Ks/2r to calculate the date of the replication event, where “r” is the neutral substitution rate. The neutral replacement rate used in the current study is 2.6 × 10^−9^ ^45^.

### Gene structure and analyses of conserved motifs

The cDNA sequences and intron/exon lengths in TCP genes were obtained from the *G*. *barbadense* database, and the GbTCP gene family was analyzed online using the Gene Structure Display Server (http://gsds.cbi.pku.edu.cn/)^46^. Conserved motifs were predicted in the *G*. *barbadense* TCP family of proteins using the MEME website (http://meme-suite.org/index.html)^47^. The parameters were set to: “any” motif repeat number, 6 to 100 motif width, and maximum number of motifs: 20. In addition, we use the InterProScan4 program to annotate the identified motif^48^.

### RNA extraction and qRT-PCR

Total RNA was extracted from *G*. *barbadense* fiber tissues on days 0, 5, 10, 15, 20, 25, 30, and 35 using an RNAprep Pure Plant Kit (Tiangen, http://www.tiangen.com). RNAs were processed to remove genomic DNA. A Colibri Microvolume Spectrometer (Titertek-Berthold, http://www.titertek-berthold.com) was used to assess the concentration and quality of RNA. First-strand cDNA was synthesized using a Transcriptor First Strand cDNA Synthesis Kit (Thermo Scientific, http://www.Thermo.com) with 2 µg total RNA. According to the sequences of 75 genes from the TCP of the *G*. *barbadense*, 75 primer pairs (Supplementary Dataset 2) were designed using Primer Express 3.0.1. The annealing temperature was between 58 °C and 60 °C for qRT-PCR. The *G*. *barbadense* UBQ7 gene was used as a reference gene, using 20 μL per reaction. The reactions included 1.5 μL cDNA, 10 μL 2 × PowerUp^TM^SYBR^TM^ Green Master Mix (Applied Biosystems, USA), 0.4 μL each of upstream and downstream primers, and 7.7 μL RNase-Free ddH_2_O. Two replicate samples of each period were subjected to three biological replicates using an ABI 7500 Fast Real-Time PCR instrument (Applied Biosystems, USA). Amplification parameters were as follows: activation at 50 °C for 2 min, pre-denaturation at 95 °C for 2 min, denaturation at 95 °C for 15 s, and annealing at 60 °C for 1 min for 40 cycles. The data were quantitatively analyzed using the 2^−△△CT^ method^49^ and were processed using Microsoft Excel 2010 software.

## Electronic supplementary material

Supplementary Information

Supplementary Dataset 1

Supplementary Dataset 2

Supplementary Dataset 3

Supplementary Dataset 4

Supplementary Dataset 5

Supplementary Dataset 6

## References

[1] Cubas P, Lauter N, Doebley J, Coen E (1999). The TCP domain: a motif found in proteins regulating plant growth and development. The Plant Journal.

[2] Kosugi S, Ohashi Y (1997). PCF1 and PCF2 specifically bind to cis elements in the rice proliferating cell nuclear antigen gene. The Plant Cell.

[3] Martíntrillo M, Cubas P (2010). TCP genes: a family snapshot ten years later. Trends in Plant Science.

[4] Doebley J, Stec A, Hubbard L (1997). The evolution of apical dominance in maize. Nature.

[5] Luo D, Carpenter R, Vincent C, Copsey L, Coen E (1996). Origin of floral asymmetry in Antirrhinum. Nature.

[6] Palatnik JF (2003). Control of leaf morphogenesis by microRNAs. Nature.

[7] Costa MMR, Fox S, Hanna AI, Baxter C, Coen E (2005). Evolution of regulatory interactions controlling floral asymmetry. Development.

[8] Broholm SK (2008). TCP domain transcription factor controls flower type specification along the radial axis of the Gerbera (Asteraceae) inflorescence. Proceedings of the National Academy of Sciences.

[9] Navaud O, Dabos P, Carnus E, Tremousaygue D, Hervé C (2007). TCP transcription factors predate the emergence of land plants. Journal of molecular evolution.

[10] Reeves PA, Olmstead RG (2003). Evolution of the TCP gene family in Asteridae: cladistic and network approaches to understanding regulatory gene family diversification and its impact on morphological evolution. Molecular Biology and Evolution.

[11] Kölsch A, Gleissberg S (2006). Diversification of CYCLOIDEA‐like TCP Genes in the Basal Eudicot Families Fumariaceae and Papaveraceae s. str. Plant Biology.

[12] Citerne HL, Luo D, Pennington RT, Coen E, Cronk QC (2003). A phylogenomic investigation of CYCLOIDEA-like TCP genes in the Leguminosae. Plant Physiology.

[13] Li S (2015). The Arabidopsis thaliana TCP transcription factors: a broadening horizon beyond development. Plant signaling & behavior.

[14] Yao X, Ma H, Wang J, Zhang D (2007). Genome‐Wide Comparative Analysis and Expression Pattern of TCP Gene Families in Arabidopsis thaliana and Oryza sativa. Journal of Integrative Plant Biology.

[15] Ma X (2016). Genome-wide Identification of TCP family transcription factors from populus euphratica and their involvement in leaf shape regulation. Scientific reports.

[16] Parapunova V (2014). Identification, cloning and characterization of the tomato TCP transcription factor family. BMC plant biology.

[17] Shi P (2016). Genome-wide identification and expression analysis of the ClTCP transcription factors in Citrullus lanatus. BMC plant biology.

[18] De Paolo S, Gaudio L, Aceto S (2015). Analysis of the TCP genes expressed in the inflorescence of the orchid Orchis italica. Scientific reports.

[19] Francis A (2016). Comparative phylogenomic analysis provides insights into TCP gene functions in Sorghum. Scientific reports.

[20] Ma J (2014). Genome-wide identification and expression analysis of TCP transcription factors in Gossypium raimondii. Scientific reports.

[21] Ma J (2016). Comprehensive analysis of TCP transcription factors and their expression during cotton (Gossypium arboreum) fiber early development. Scientific reports.

[22] Li W (2017). Genome-wide identification and characterization of TCP transcription factor genes in upland cotton (Gossypium hirsutum). Scientific Reports.

[23] Schommer C (2008). Control of jasmonate biosynthesis and senescence by miR319 targets. PLoS biology.

[24] Hervé C (2009). *In vivo* interference with attcp20 function induces severe plant growth alterations and deregulates the expression of many genes important for development. Plant Physiology.

[25] Pagnussat GC (2005). Genetic and molecular identification of genes required for female gametophyte development and function in arabidopsis. Development.

[26] Takeda T (2006). Rna interference of the arabidopsis putative transcription factor tcp16 gene results in abortion of early pollen development. Plant Molecular Biology.

[27] Prunedapaz JL, Breton G, Para A, Kay SA (2009). A functional genomics approach reveals che as a component of the arabidopsis circadian clock. Science.

[28] Alghazi Y, Bourot S, Arioli T, Dennis ES, Llewellyn DJ (2009). Transcript profiling during fiber development identifies pathways in secondary metabolism and cell wall structure that may contribute to cotton fiber quality. Plant & Cell Physiology.

[29] Wang K (2012). The draft genome of a diploid cotton Gossypium raimondii. Nature genetics.

[30] Li F (2014). Genome sequence of the cultivated cotton Gossypium arboreum. Nature genetics.

[31] Liu X (2015). Gossypium barbadense genome sequence provides insight into the evolution of extra-long staple fiber and specialized metabolites. Scientific Reports.

[32] Wang MY (2013). The cotton transcription factor TCP14 functions in auxin-mediated epidermal cell differentiation and elongation. Plant physiology.

[33] Cannon SB, Mitra A, Baumgarten A, Young ND, May G (2004). The roles of segmental and tandem gene duplication in the evolution of large gene families in Arabidopsis thaliana. BMC plant biology.

[34] Hu W, Hu G, Han B (2009). Genome-wide survey and expression profiling of heat shock proteins and heat shock factors revealed overlapped and stress specific response under abiotic stresses in rice. Plant Science.

[35] Hao J (2012). GbTCP, a cotton TCP transcription factor, confers fibre elongation and root hair development by a complex regulating system. Journal of Experimental Botany.

[36] Mao Y (2014). MicroRNA319a-targeted Brassica rapa ssp. pekinensis TCP genes modulate head shape in chinese cabbage by differential cell division arrest in leaf regions. Plant physiology.

[37] Kieffer M, Master V, Waites R, Davies B (2011). TCP14 and TCP15 affect internode length and leaf shape in Arabidopsis. The Plant Journal.

[38] Thompson JD, Gibson TJ, Plewniak F, Jeanmougin F, Higgins DG (1997). The clustal_x windows interface: flexible strategies for multiple sequence alignment aided by quality analysis tools. Nucleic Acids Research.

[39] Tamura K, Stecher G, Peterson D, Filipski A, Kumar S (2013). Mega6: molecular evolutionary genetics analysis version 6.0. Molecular Biology & Evolution.

[40] Wang Y (2012). MCScanX: a toolkit for detection and evolutionary analysis of gene synteny and collinearity. Nucleic acids research.

[41] Krzywinski M (2009). Circos: an information aesthetic for comparative genomics. Genome Research.

[42] Hu R (2010). Comprehensive analysis of NAC domain transcription factor gene family in Populus trichocarpa. BMC plant biology.

[43] Librado P, Rozas J (2009). DnaSPv5: a software for comprehensive analysis of DNA polymorphism data. Bioinformatics.

[44] Yang X, Tuskan GA (2006). Divergence of the Dof gene families in poplar, Arabidopsis, and rice suggests multiple modes of gene evolution after duplication. Plant physiology.

[45] Zhang T (2015). Sequencing of allotetraploid cotton (Gossypium hirsutum L. acc. TM-1) provides a resource for fiber improvement. Nature biotechnology.

[46] Hu B (2014). GSDS 2.0: an upgraded gene feature visualization server. Bioinformatics.

[47] Bailey TL (2009). MEME SUITE: tools for motif discovery and searching. Nucleic acids research.

[48] Quevillon, E. *et al*. Interproscan: protein domains identifier. Nucleic Acids Research, 33 (Web Server issue), 116–20 (2005). 10.1093/nar/gki442 PMC1160203 15980438

[49] Livak KJ, Schmittgen TD (2001). Analysis of relative gene expression data using real-time quantitative PCR and the 2−ΔΔCT method. methods.

