# Supplementary material for: Genome-wide identification and expression analyses of TCP transcription factor genes in Gossypium barbadense
Source: Sci Rep. 2018 Sep 28;8:14526. doi: 10.1038/s41598-018-32626-5 (PMC6162280; doi:10.1038/s41598-018-32626-5)
Supplement: Supplementary file 1 — Supplementary Information [file 41598_2018_32626_MOESM1_ESM.docx]

Supplementary Information

**Genome-wide identification and expression analyses of TCP transcription factor genes in *Gossypium barbadense***

Kai Zheng^a, 1^, Zhiyong Ni ^a, 1^, Yanying Qu^1^, Yongsheng Cai^1^, Zhaoen Yang^1^, Guoqing Sun^2^**^＊^**, Quanjia Chen^1^**^＊^**

^1^ College of Agronomy, Xinjiang Agricultural University, Urumqi 830052, PR China

^2^ Biotechnology Research Institute, Chinese Academy of Agricultural Sciences, Beijing 100081, PR China

**^＊^**To whom correspondence should be addressed.

Quanjia Chen

E-mail: chqjia@126.com

Tel (Fax): (+86) 991 876 2412

College of Agronomy, Xinjiang Agricultural University, No.311 Nongda East Street, Shayibake District, Urumqi 830052, PR China

Guoqing Sun

E-mail: sunguoqing02@caas.cn

Biotechnology Research Institute, Chinese Academy of Agricultural Sciences, No. 12 Zhongguancun South Street, Haidian District, Beijing 100081, PR China

a. Kai Zheng and Zhiyong Ni contributed equally to this work

**Supplementary Table.1** Correlation of TCP Genes Expression in Different Developmental Stages of Cotton

**Supplementary Table.2**

**Supplementary Fig. 1** Expression analysis of the upland cotton GhTCP genes in fiber development using qRT-PCR.

**Supplementary Fig.2** Phylogenetic analysis of the GbTCP gene family from *Gossypium arboreum*, *G*. *raimondii*, *G*. *barbadense*, *Theobroma cacao*, *G*. *hirsutum*, *Arabidopsis thaliana*, *Oryza sativa*, *Sorghum bicolor,* and *Zea mays.*

**Supplementary Fig. 3** Sequence logo of the motifs of GbTCPs.

| Item | Xinhai25 | Xinluzhong36 | Ashmon |
| --- | --- | --- | --- |
| Xh25 | 1 | 0.468^*^ | 0.379 |
| Xinluzhong36 | 0.468^*^ | 1 | 0.373 |
| Ashmon | 0.379 | 0.373 | 1 |

Note:* and ** represent 0.05 and 0.01 significance levels, respectively.

**Supplementary Table.1** Correlation of six TCP Genes Expression in different developmental stages of cotton.

| **Orthlog gene name in At subgenome(Chromosome)** | **Corresponding Orthlog gene name in Dt subgenome (Chromosome)** |
| --- | --- |
| GbTCP7_A01 | GbTCP6_D01 |
| GbTCP14_A03 | GbTCP13_D02 |
| GbTCP9_A04 | GbTCP8_D05 |
| GbTCP21_A04 | GbTCP19_D04 |
| GbTCP4_A05 | NA |
| GbTCP71_A05 | GbTCP20_D04 |
| GbTCP33_A05 | GbTCP34_D04 |
| GbTCP39_A07 | GbTCP38_D07 |
| GbTCP50_A07 | NA |
| GbTCP1_A07 | NA |
| GbTCP35_A09 | NA |
| GbTCP63_A09 | GbTCP64_D07 |
| GbTCP62_A09 | GbTCP65_D12 |
| GbTCP56_A09 | GbTCP55_ D09 |
| GbTCP57_A09 | GbTCP55_ D09 |
| GbTCP72_A10 | GbTCP5_ D10 |
| GbTCP30_A10 | GbTCP12_ D10 |
| GbTCP52_A11 | GbTCP51_ D11 |
| GbTCP36_A11 | GbTCP37_ D11 |
| GbTCP25_A11 | GbTCP26_ D08 |
| GbTCP27_A11 | NA |
| GbTCP42_A12 | GbTCP40_ D12/ GbTCP41_ D07 |
| GbTCP18_A12 | GbTCP17_ D12 |
| GbTCP75_A12 | GbTCP12_ D10 |
| GbTCP74_A12 | GbTCP43_ D12 |
| GbTCP32_A12 | GbTCP31_ D12 |
| GbTCP11_A12 | GbTCP10_ D12 |
| GbTCP59_A12 | NA |
| GbTCP54_A12 | GbTCP53_ D12 |
| GbTCP15_A13 | NA |
| GbTCP49_A13 | GbTCP45_ D13 |
| GbTCP48_A13 | GbTCP44_ D12 |
| GbTCP47_A13 | GbTCP46_ D13 |
| GbTCP67_A13 | GbTCP68_ D13 |
| GbTCP66_A13 | GbTCP69_ D13 |
| NA | GbTCP73_D04 |
| NA | GbTCP70_D01 |
| GbTCP2_ Scaffold 17189.2.0 | NA |
| GbTCP23_ Scaffold 6358.4.0 | NA |
| GbTCP24_ Scaffold 6358.5.0 | NA |
| GbTCP28_ Scaffold 10005.2.0 | NA |
| GbTCP29_ Scaffold 10005.3.0 | NA |
| GbTCP58_Scaffold 3276 | NA |
| GbTCP16_ Scaffold 6022 | NA |
| GbTCP22_Scaffold 6358.6 | NA |
| GbTCP3_ Scaffold 13071 | NA |

**Supplementary Table.2** Classify GbTCP genes on the basis of A and D sub genomes





**Supplementary Fig. 1** Expression of 6 GhTCP genes in various stages of upland cotton fiber development. The X-axis represents fiber samples from different growth periods, and the Y-axis represents the relative expression level of GhTCP gene. Error bars represent the standard deviation of three replicates.


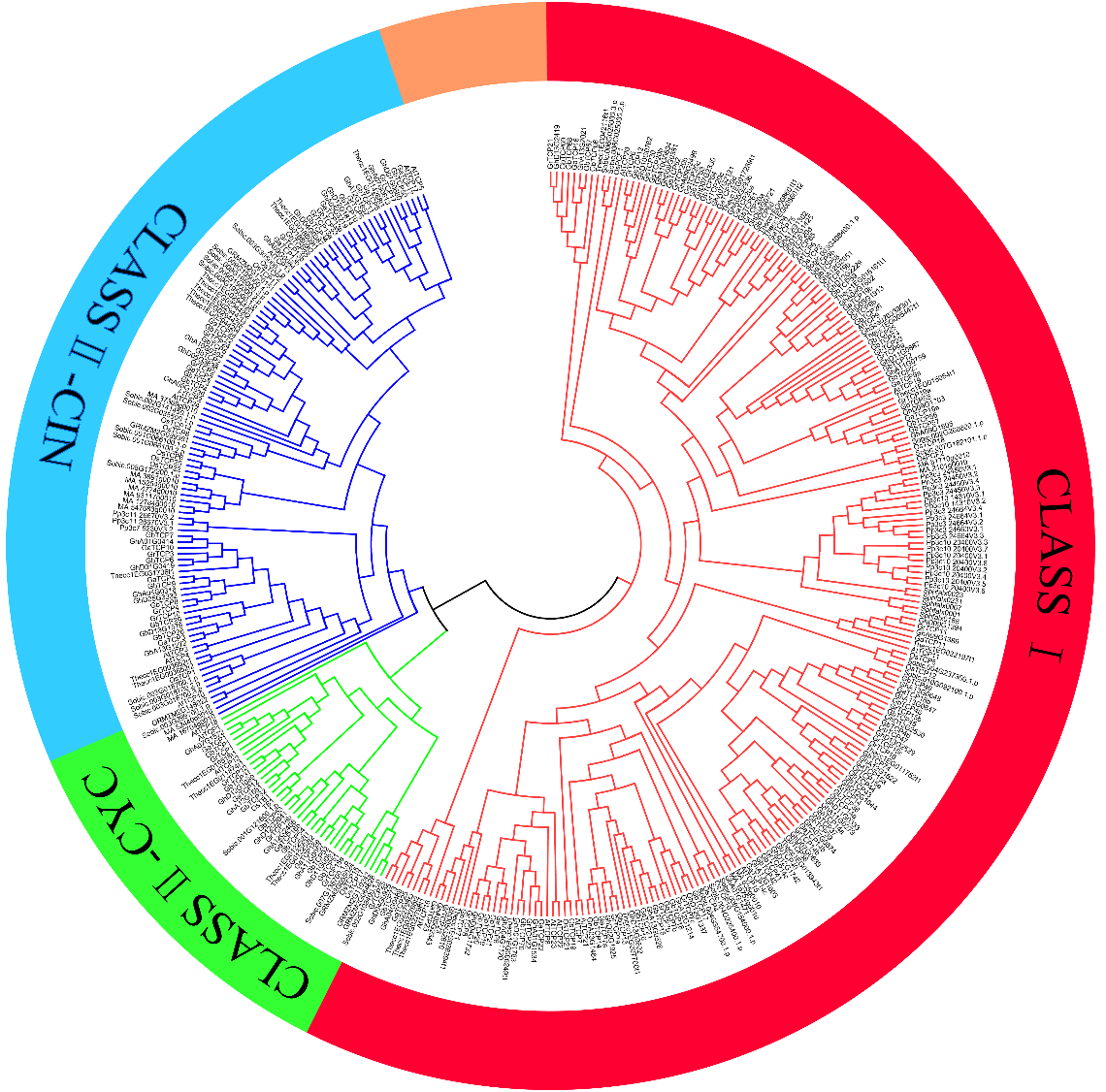


**Supplementary Fig. 2** Phylogenetic tree of TCP proteins from *Gossypium arboreum*, *G*. *raimondii*, *G*. *barbadense*, *Theobroma cacao*, *G*. *hirsutum*, *Arabidopsis thaliana*, *Oryza sativa*, *Sorghum bicolor*, and *Zea mays*, *Picea abies* , *Sphagnum fallax* and *Physcomitrella patens* using the MEGA6.0 software with the maximum likelihood (ML) method with 1,000 resampling replicates. Each GbTCP subfamily is indicated by a specific color.


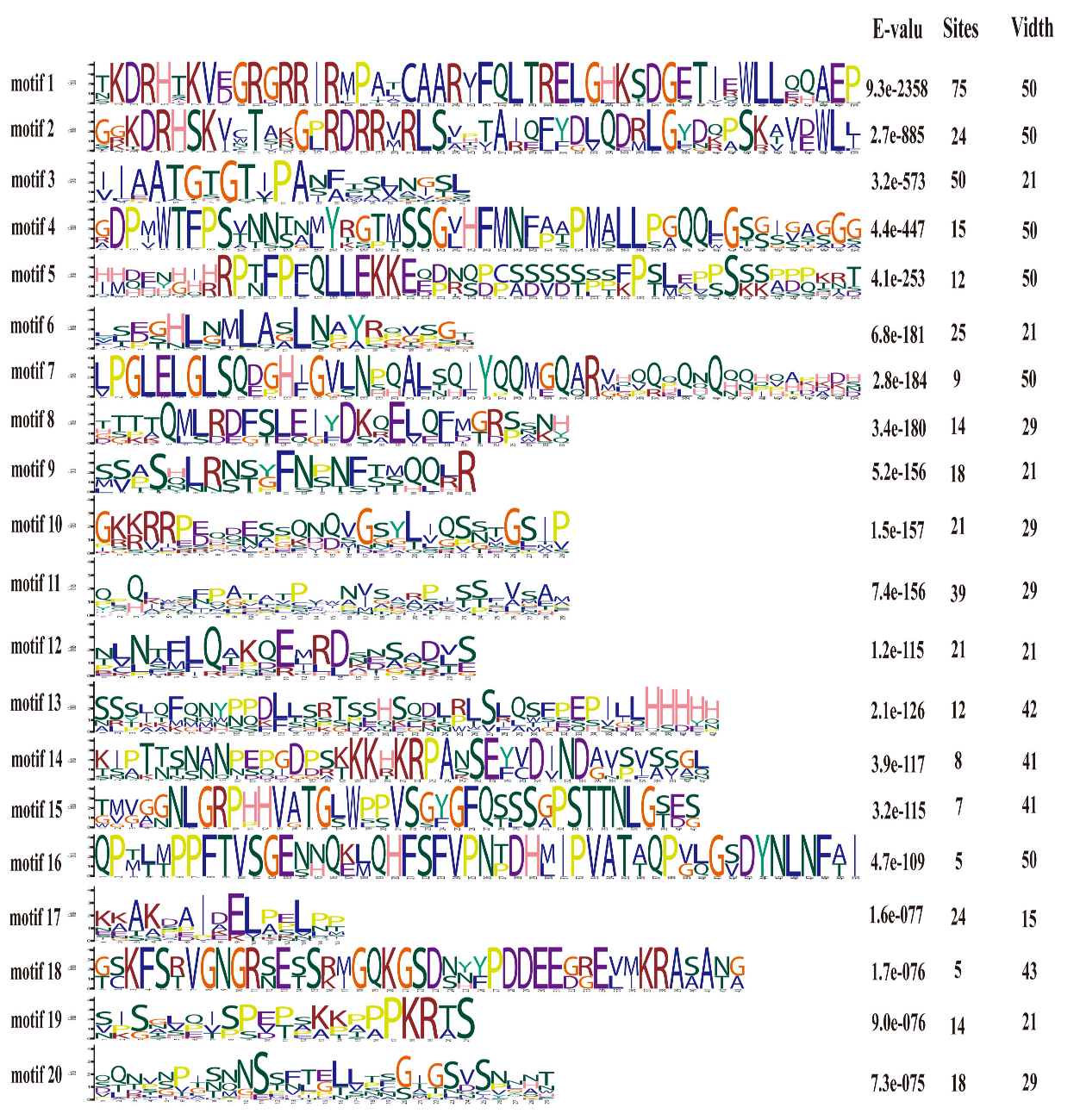


**Supplementary Fig. 3 Sequence logo of the motifs of GbTCPs.**
